# Revolutionizing Home Safety and Independence for Older Adults With the Augmented Reality Home Assessment Tool

**DOI:** 10.1093/geroni/igaf122.994

**Published:** 2025-12-31

**Authors:** Emily Thompson, Jung-hye Shin, Kevin Ponto, Beth Fields

**Affiliations:** University of Wisconsin–Madison, Madison, Wisconsin, United States; University of Wisconsin–Madison, Madison, Wisconsin, United States; University of Wisconsin–Madison, Madison, Wisconsin, United States; University of Wisconsin–Madison, Madison, Wisconsin, United States

## Abstract

The number of older adults is rising due to medical advancements, with one in four U.S. households including older adults in 2019. Many of these older adults have a strong desire to remain in their homes safely and independently for as long as possible. However, fewer than 1% of homes are equipped to accommodate their needs, increasing their risk of falls and healthcare service use. The Augmented Reality Home Assessment Tool (ARHAT), a mobile-based app, addresses this gap by using 3D scanning and augmented reality capabilities to assess home environments. ARHAT has been accepted and deemed appropriate by key end users, including occupational therapists, housing professionals, and older adults, for identifying and addressing functional limitations and barriers in home environments. In partnership with a community-based organization, this study aims to evaluate the feasibility of implementing ARHAT and its impact on reducing fear of falling and healthcare service use among 75 community-dwelling older adults in the Midwest. Descriptive and inferential statistics will be conducted using STATA software. Preliminary data to be presented include recruitment, retention, readiness to implement, adherence, demographics, fear of falling, and healthcare service use. “What Matters” to older adults is the ability to age in their home environment safely and independently. ARHAT aligns with this goal by providing a practical, acceptable, and appropriate tool for enhancing home safety and supporting aging in place.

